# Integration of Metabolomics and Gene Expression Profiling Elucidates IL4I1 as Modulator of Ibrutinib Resistance in ABC-Diffuse Large B Cell Lymphoma

**DOI:** 10.3390/cancers13092146

**Published:** 2021-04-29

**Authors:** Fouad Choueiry, Satishkumar Singh, Anuvrat Sircar, Georgios Laliotis, Xiaowei Sun, Evangelia Chavdoula, Shiqi Zhang, JoBeth Helmig-Mason, Amber Hart, Narendranath Epperla, Philip Tsichlis, Robert Baiocchi, Lapo Alinari, Jiangjiang Zhu, Lalit Sehgal

**Affiliations:** 1 Department of Human Sciences, The Ohio State University, Columbus, OH 43210, USA; choueiry.2@buckeyemail.osu.edu (F.C.); sunx5@miamioh.edu (X.S.); Zhang.6517@osu.edu (S.Z.); 2 Division of Hematology, Department of Internal Medicine, The Ohio State University, Columbus, OH 43210, USA; Satishkumar.Singh@osumc.edu (S.S.); Anuvrat.Sircar@osumc.edu (A.S.); JoBeth.Helmig-Mason@osumc.edu (J.H.-M.); hart.689@buckeyemail.osu.edu (A.H.); narendranath.epperla@osumc.edu (N.E.); Robert.Baiocchi@osumc.edu (R.B.); Lapo.Alinari@osumc.edu (L.A.); 3 James Comprehensive Cancer Center, The Ohio State University, Columbus, OH 43210, USA; Georgios.Laliotis@osumc.edu (G.L.); Evangelia.Chavdoula@osumc.edu (E.C.); Philip.Tsichlis@osumc.edu (P.T.); 4 Department of Cancer Biology and Genetics, The Ohio State University, Columbus, OH 43210, USA

**Keywords:** DLBCL, lymphoma, transcriptomic, metabolomics, drug resistance, ibrutinib, amino-acid metabolism, metabolic reprogramming

## Abstract

**Simple Summary:**

In this study, we present a workflow to understand the modulator of ibrutinib resistance in ABC diffuse large B cell lymphoma by integrating Metabolomics and Gene expression profiling as shown in the graphical abstract. We performed an untargeted metabolomics analysis using a Q-Exactive high-resolution mass spectrometer to dissect the metabolic reprogramming associated with acquired ibrutinib resistance in paired ibrutinib-sensitive and ibrutinib-resistant DLBCL cell lines. Further, we identified common denominators, integrating metabolome and transcriptome data, confirming clinical significance, integrating pathways, and identifying the candidate gene driving ibrutinib resistance and metabolic reprogramming. Our work demonstrates that a multi-omics approach can be a robust and impartial strategy to uncover genes and pathways that cause metabolic deregulation in cancer cells.

**Abstract:**

Diffuse large B-cell lymphoma (DLBCL) is the most common non-Hodgkin lymphoma (NHL). B-cell NHLs rely on Bruton’s tyrosine kinase (BTK) mediated B-cell receptor signaling for survival and disease progression. However, they are often resistant to BTK inhibitors or soon acquire resistance after drug exposure resulting in the drug-tolerant form. The drug-tolerant clones proliferate faster, have increased metabolic activity, and shift to oxidative phosphorylation; however, how this metabolic programming occurs in the drug-resistant tumor is poorly understood. In this study, we explored for the first time the metabolic regulators of ibrutinib-resistant activated B-cell (ABC) DLBCL using a multi-omics analysis that integrated metabolomics (using high-resolution mass spectrometry) and transcriptomic (gene expression analysis). Overlay of the unbiased statistical analyses, genetic perturbation, and pharmaceutical inhibition was further used to identify the key players contributing to the metabolic reprogramming of the drug-resistant clone. Gene-metabolite integration revealed interleukin four induced 1 (IL4I1) at the crosstalk of two significantly altered metabolic pathways involved in producing various amino acids. We showed for the first time that drug-resistant clones undergo metabolic reprogramming towards oxidative phosphorylation and are modulated via the BTK-PI3K-AKT-IL4I1 axis. Our report shows how these cells become dependent on PI3K/AKT signaling for survival after acquiring ibrutinib resistance and shift to sustained oxidative phosphorylation; additionally, we outline the compensatory pathway that might regulate this metabolic reprogramming in the drug-resistant cells. These findings from our unbiased analyses highlight the role of metabolic reprogramming during drug resistance development. Our work demonstrates that a multi-omics approach can be a robust and impartial strategy to uncover genes and pathways that drive metabolic deregulation in cancer cells.

## 1. Introduction

Diffuse large B-cell lymphoma (DLBCL) is the most common non-Hodgkin lymphoma (NHL) and is clinically heterogeneous: 30–40% of patients respond well to current therapy and have prolonged survival, whereas the remainder succumb to the disease [1,2,3,4,5,6]. Patients with DLBCL can be classified into either the activated B-cell (ABC), the germinal center B-cell (GCB), or unclassified molecular subtype according to the cell of origin determined by gene expression profiling and are characterized by distinct gene mutation signatures [3,4,6,7]. The majority of patients with DLBCL (~60%) are cured with rituximab, cyclophosphsphomide, hydroxydaunomycin, oncovin, and prednisone cocktail (R-CHOP) chemotherapy, but nearly one in three patients will develop relapsed/refractory disease [6,8,9]. Patients with relapsed/refractory disease have an increased morbidity and mortality rate despite treatment with salvage therapies. Following multi-agent chemotherapy, the ABC molecular subtype has a lower survival rate compared to the GCB subtype [10,11]. Because ABC-DLBCL has chronically active B-cell receptor signaling, several components of these signaling pathways are attractive therapeutic targets [12]. Bruton’s tyrosine kinase (BTK) drives the B-cell receptor-signaling cascade, which leads to the activation of NF-kB and other targets [13,14]. The orally administered, bioavailable BTK inhibitor ibrutinib has been FDA-approved to treat patients with relapsed mantle cell lymphoma (MCL), Waldenstrom’s macroglobulinemia, and chronic lymphocytic leukemia (CLL), including those harboring 17p deletion [15,16]. In a phase I/II clinical trial of relapsed/refractory DLBCL, 37% of ABC-DLBCL patients responded to ibrutinib [12]. Despite these encouraging results, the ibrutinib treatment produces variable or incomplete responses and leads to drug resistance and genetic alterations stemming from unknown causes [12,17]. Recently, Schmitz et al. found that responses to ibrutinib were persistent in ABC tumors with both a mutation in CD79B, encoding a B-cell receptor subunit, and the MYD88L265P mutation, a finding that suggests that tumor genotype could influence response [12]. Molecular subsets of lymphomas possessing distinct metabolic signatures provide the unique opportunity to investigate the role of metabolic changes in disease progression and response to chemotherapeutics. These lymphomas are categorized based on cellular reliance on increased mitochondrial oxidative phosphorylation versus cellular dependence on B-cell receptor signaling [18,19,20]. For instance, a comparison of the genetic signatures across DLBCLs using genome-wide arrays and multiple clustering algorithms captured tumor-intrinsic distinctions in three clusters [19] (a) B-cell receptor (BCR) /proliferation cluster (BCR-DLBCL) displaying up-regulation of genes encoding BCR signaling components, (b) the oxidative phosphorylation cluster (OXPHOS DLBCL), and (c) the host response cluster (HR). This multi-omics approach has also been used to study metabolic deregulations, paving the way to identify metabolic vulnerabilities in cancer [18].

Metabolomics can identify metabolic changes or anomalies in biological systems and deliver qualitative and quantitative information about the steady-state prevalence of substrates from various metabolic pathways. Thus, metabolomics studies can provide an overview of the metabolic deregulations affecting cellular physiology in disease. The metabolome reflects the phenotypic outcome of genomic, transcriptomic, proteomic, and environmental influences on the molecular state of the cell. For this reason, the metabolome can be considered the end readout of ‘multi-omics’-driven cell physiology. In the context of cancer biology, the activation of oncogenes by genetic mutation or deregulated gene expression can drive a cancer cell into a physiological state in which cell metabolism is effectively reprogrammed. A genomics approach can identify genetic mutations that can derail the cell’s physiology. In contrast, a transcriptomic approach can identify RNA species whose abnormal expression contributes to an altered physiological state of the cancer cell. In summary, a multi-omics approach provides a multi-layered insight into how upstream changes (such as those observed in genomic, transcriptomic, and proteomic profiles) exert changes on downstream metabolic indicators of the cell’s physiological state. 

Understanding energy utilization pathways and the metabolic deregulation within DLBCL and other similarly heterogeneous groups of tumors have not been fully elucidated. Metabolic active DLBCL tumors do not rely upon/display an operational/functional BCR signaling [21], suggesting that the tumors that lack BCR signaling are more prone to metabolic deregulation. Recently we have shown that BCR signaling-dependent ABC-DLBCL cells lack B-cell signaling upon acquiring ibrutinib resistance [22]. We hypothesized that the acquired ibrutinib-resistant ABC-DLBCL that lacks BCR signaling is more likely to exhibit metabolic alteration. Identification of the metabolic landscape in the ibrutinib resistant molecular subset can be a potential therapeutic target. Here, we conducted an untargeted metabolomics analysis using a Q-Exactive high-resolution mass spectrometer to dissect the metabolic reprogramming associated with acquired ibrutinib resistance in paired ibrutinib-sensitive and ibrutinib-resistant DLBCL cell lines [22]. Further, we identified common denominators, integrating metabolome and transcriptome data, indicating candidate molecular pathways driving ibrutinib resistance in DLBCL.

## 2. Materials and Methods 

### 2.1. Cell Culture/Ibrutinib Resistance

DLBCL cell lines HBL1 and TMD8 were cultured in RPMI-1640 media supplemented with 10% fetal bovine serum as described previously [22]. For the glucose and galactose assay, either glucose-free Dulbecco’s modified Eagle medium (RPMI-1640; Thermo Fisher Scientific, Waltham, MA, USA) supplemented with 10 mM glucose, 10% FBS, 50 units/mL of penicillin and streptomycin, and 1 mM of pyruvate (glucose: OXPHOS-independent conditions) or glucose-free RPMI-1640 medium (Thermo Fisher Scientific) supplemented with 10 mM galactose, 10% FBS, 50 units/mL of penicillin and streptomycin, and 1 mM of pyruvate (galactose: OXPHOS-competent enriched conditions). Sugar concentrations of 10 mM were chosen to mimic physiological sugar levels (glucose, 10 mM) and avoid potential biological artifacts mediated by supraphysiological sugar levels. Cultures were routinely tested for mycoplasma, and cell line identities were confirmed by short tandem repeat (STR) testing. The BTK inhibitor ibrutinib (PCI-32765) was purchased from Selleckchem. Ibrutinib-resistant cells were generated as described previously. Briefly, wild-type (WT) cell lines were perpetually cultured with incremental doses of ibrutinib for 8–10 months. Cells were passaged routinely upon confluency. However, cells passaged to less than 20 passages were utilized for experiments after a week of drug-free culture. When cultured in the absence of ibrutinib, all cell lines generated in this study exhibited steady ibrutinib resistance. The wild-type BTK construct was cloned in the base vector (Lenti-X Expression System Version EF1-a; Takara Bio, Mountain view, CA, USA). Stable over-expression of BTK in ibrutinib-resistant DLBCL cells was performed using lentiviruses expressing human wild-type BTK, as described previously [22]. 

### 2.2. Proliferation Assay and Doubling Time

Proliferation assays described here used the new passage of the cells. Absorbance was recorded after adding WST-1 proliferation reagent after every 24 h of growth. Time points for calculating doubling time were chosen based on where the growth curve was linear when the log of absorbance was a plot against time, indicating optimal growth. Based on Beer–Lambert’s law, absorbance (A) equals KLC, where k = proportionality constant, l = path length, and c = concentration. If k and l are constant in starting and ending time points, log (C2/C1) equals log (A2/A1). Further, the doubling time can be calculated using the following formula: Doubling Time = duration ∗ log(2)/log(FinalConcentration)—log(InitalConcentration), or using an online tool (http://www.doubling-time.com/compute.php, accessed on 13 March 2021).

### 2.3. IC50

To quantify surviving and/or proliferating cells, a WST-1 cell proliferation assay kit (MK400, Takara Bio) was used according to the manufacturer’s instructions. In each experiment, 10,000 cells were seeded into each well of a 96-well culture dish, and ibrutinib was added the following day. Average relative absorption (OD450-OD690) was measured 72 h after the addition of ibrutinib to estimate the number of metabolically active cells. The IC-50 was calculated using the Quest Graph™ IC50 Calculator. (AAT Bioquest, Inc, https://www.aatbio.com/tools/ic50-calculator, accessed on 20 April 2021).

### 2.4. Gene Expression Studies

Ibrutinib-resistant DLBCL cell line HBL1-R and parental line HBL1 were used in triplicate for the gene expression studies. Briefly, total RNA from each sample was quantified using the NanoDrop ND-1000 spectrophotometer. RNA integrity was assessed by standard denaturing agarose gel electrophoresis. For microarray analyses, total RNA from each sample was amplified and transcribed into fluorescent complementary RNA using the manufacturer’s Quick Amp Gene Expression Labeling Protocol, Version 5.7.9 (Agilent Technologies, Santa Clara, CA, USA). The labeled complementary RNAs were hybridized onto a whole human genome oligo microarray (4 3 44K; Agilent Technologies). After washing the slides, the arrays were scanned using the Agilent microarray scanner G2505C. Agilent’s Feature Extraction software (version 11.0.1.1) was used to analyze array images. Quantile normalization and subsequent data processing were performed using the GeneSpring GX v12.1 software (Agilent Technologies). After quantile normalization of the raw data, genes that had flags in at least 3 of 24 samples were chosen for further data analyses. Differentially expressed genes were identified through fold-change and volcano filtering. Agilent’s pathway and Gene Ontology analyses were applied to determine the roles that these differentially expressed genes play in these biological pathways or Gene Ontology terms. Finally, a Venn diagram was generated to show the distinguishable gene expression profiles among samples. We deposited the corresponding raw data into the Gene Expression Omnibus data repository under accession number GSE138126.

### 2.5. Immunoblotting

For immunoblotting, cells were harvested and lysed in ice-cold radio-immunoprecipitation lysis buffer (Cell Signaling Technology, Danvers, MA, USA) containing a protease inhibitor cocktail (Roche). Equal amounts of proteins were resolved using sodium dodecyl sulfate-polyacrylamide gel electrophoresis, transferred to PVDF membrane (Bio-Rad, Hercules, CA, USA), and analyzed with the following specific primary antibodies: anti-BTK (D3H5), anti-IL4I1 (ab-222101, Abcam), TCA CST kit (#47767), and the a-actinin CST (#6487) and horseradish peroxidase-conjugated anti-β-actin (A3854; Millipore Sigma, St. Louis, MO, USA) using Bio-Rad (Hercules, CA, USA) ChemiDoc Imaging system (#12003153).

### 2.6. Quantitative qPCR

Total RNA was isolated with the Qiagen RNeasy Plus Mini Kit following the manufacturer’s instructions. After DNase treatment, cDNA was synthesized using a SuperScript™ III First-Strand Synthesis System (Invitrogen) using 1 μg of total RNA. Quantitative-PCR was carried out on a CFX96 real-time PCR system (Bio-Rad) using SYBR green PCR master mix (Applied Biosystems). Total RNA from each sample was normalized to β-actin followed by calculating average relative quantity (RQ). The following primers were used: IL4I1 (forward primer: CGCCCGAAGACATCTACCAG, reverse primer: GATATTCCAAGAGCGTGTGCC), GOT1 (forward primer: AGTCTTTGCCGAGGTTCCG, reverse primer: GTGCGATATGCTCCCACTC), GOT2 (forward primer: GACCAAATTGGCATGTTCTGT, reverse primer: CGGCCATCTTTTGTCATGTA), ALDH7A1 (forward primer: CAACGAGCCAATAGCAAGAG, reverse primer: GCATCGCCAATCTGTCTTAC), LAP3 (forward primer: TCGGCAAAGCTCTATGGAAGT, reverse primer: GCGTCATCTCATTGGCTGG) and ALDH9A1 (forward primer: TGGAGTCAAAAATCTGGCATGG, reverse primer: AGTAGCAATTTCATCCTCCCGT).

### 2.7. Metabolite Extraction

Intracellular metabolites from each biological replicate were extracted using cold methanol-based extraction. Cells were harvested and counted, and 1 × 10^6^ cells were aliquoted in triplicate for metabolite extraction. Cells were washed using cold PBS before the addition of 250 μL of methanol (LCMS-grade). Internal standards containing ^13^C and ^15^N labeled amino acids mix (1.2 mg/mL methanol) were added to the samples at a volume of 50 μL. Cells were homogenized for 2 min before incubating at −20 °C for 20 min to extract polar metabolites from the cells. Cells were pelleted, and 150 μL of supernatant was transferred to an LC-MS vial. A pooled quality control (QC) sample was collected by aliquoting an equal volume of supernatants from all biological samples into a separate vial and homogenized by vortexing. Sample vials were placed in the autosampler tray at 4 °C. 

### 2.8. LC-MS/MS System

The LC-MS/MS analyses were performed on a Vanquish ultra-high-performance liquid chromatography (UHPLC) system (Thermo Scientific, Waltham MA, USA) coupled to a Q-Exactive Hybrid Quadrupole-Orbitrap Mass Spectrometer (Thermo Scientific, Waltham MA, USA). A sample volume of 5 μL was injected onto an XBridge BEH Amide XP Column, 130Å (150 mm × 2.1 mm ID, particle size 2.5 μm) (Waters Corporation, Milford, MA, USA). The column oven was maintained at 40 °C. Mobile phase A consisted of a mixture of 5 mM NH_4_Ac in ACN/H_2_O (10:90, v/v) with 0.1% acetic acid. Mobile phase B consisted of 5 mM NH_4_Ac in ACN/H_2_O (90:10, v/v) with 0.1% acetic acid. The mobile phases were delivered at a flow rate of 0.3 mL/min for a 20 min run with the following stepwise gradient for solvent B: firstly 70%; 0–5 min 30%; 5–9 min 30%; 9–11 min 70%. A divert valve was used to direct the flow to waste during the final 5 min of the run. The Q-Exactive™ was equipped with an electrospray ionization source (ESI) operated in both negative and positive ion modes to encompass a broad range of metabolite detection. The (ESI) source setting and the compound-dependent scan conditions were optimized for full-scan MS mode and ranged between 150 and 2000 m/z. The ion spray voltage was set at 4 kV with a capillary temperature of 320 °C. The sheath gas rate was set to 10 arbitrary units. Scans of 1 ms were performed at 35,000 units resolution. MS-based untargeted metabolomics was conducted in triplicate to reveal the metabolic modifications driving increased cell survival and robust proliferation in the drug-resistant phenotype. The analysis included pooled quality control samples at the beginning and end of the run and a QC followed by a blank among every ten biological sample injections. The pooled samples were also utilized for the top 10 MS/MS analyses with dynamic exclusion during the analysis for compound identification. All raw data for the MS has been deposited to MassIVE under accession number MSV000087059. 

### 2.9. Data Processing and Statistical Analysis

All raw UHPLC−MS data were imported into the automatic feature annotation and interpretation software Compound Discoverer 3.1 (Thermo Scientific, Waltham MA, USA) for metabolite identification. The MS data were searched against our in-house database containing experimentally obtained MS/MS spectra of 171 authentic analytical standards and several online databases, including KEGG (http://www.kegg.com/, accessed on 19 October 2020), the human metabolite database (http://www.hmdb.ca/, accessed on 19 October 2020), ChemSpider (http://www.chemspider.com/, accessed on 19 October 2020), and PubChem compound database (http://ncbi.nim.nih.gov/, accessed on 19 October 2020) for metabolite identification.

The collected data were then normalized to cell count per replicate, and spectra were filtered to reduce redundancy and ensure instrument reproducibility. Any metabolite with a coefficient of variance more significant than 30% was removed before further analysis. Statistical analyses, including univariate (*T*-test) and multivariate (PLS-DA) and pathway analysis, were conducted using the online resource MetaboAnalyst 4.0 [23]. The global test package was used to examine metabolites within respective pathways to determine their association with an experimental variable. Top pathways were selected based on pathway impact >0.2 and −log (p) > 2. Primary metabolites from top altered pathways were selected for further analysis. PLS-DA was used for the interpretation of the metabolic differences between the sensitive and resistant cell lines. VIP plots were generated to observe top metabolites contributing to metabolic uniqueness. 

### 2.10. Gene–Metabolite Interaction Map

Gene–metabolite interaction networks are created within MetaboAnalyst 4.0 by mapping the annotated metabolites or genes to comprehensive gene-metabolite interaction data on STITCH (‘search tool for interactions of chemicals’) [24]. Using interaction data from published peer-reviewed literature, a search algorithm can identify metabolites that immediately interact with another given compound or gene and subsequently map the nodes as direct neighbors in the network. MetaboAnalyst assesses degree centrality and betweenness centrality to determine the node importance within the network. In the interaction map, the number of connections between a node and others determines its degree of centrality. The betweenness centrality calculates the number of shortest routes going through the node. These measures allow for the identification of nodes acting as hubs in the network.

### 2.11. Early Tox Live/Dead Cell Assay

The EarlyTox Live/Dead Assay Kit (Molecular devices) was used per the manufacturer’s protocol. The kit contains two markers for live or dead cells that are suitable for use with mammalian cells. The non-fluorescent calcein AM permeates the intact cell membrane and is converted into calcein, the fluorescent form, by intracellular esterases. Live cells are stained with intense green fluorescence in the cytosol. Briefly, cells were plated at 15,000 cells per well in 10 μL in a black transparent bottom microplate. A 2× working solution of calcein-AM/EthD-III was prepared by adding calcein AM and EthD-III stock solutions to PBS for a concentration of 6 µM for each dye. A total of 100 µL of the 2× working solution was added to each assay well, resulting in a final volume of 200 µL and a final concentration of 3 µM for each dye. The plate was incubated at room temperature for 50 min or 2.5 h. It was then read from the bottom on a SpectraMax^®^ i3 Multi-Mode Microplate Reader using a preconfigured protocol in SoftMax Pro Software, and the live/dead cell ratio was plotted using GraphPad Prism. 

### 2.12. Dataset Mining and Analysis

Microarray datasets used the following: DLBCL gene expression was retrieved from different publicly available Agilent microarray datasets present on Gene Expression Omnibus (GEO) (GSE10846, GSE11318, GSE74266, GSE87371, GSE93984, GSE93985, GSE109027, GSE110376, GSE151792) by using log ratio values calculated by the authors and imported in the RStudio framework (V 3.5.2) for the gene selection (IL4I1, GOT1, ALDH7A1, LAP3, ALDH9A1, ACO2, IDH2, MCP1, MCP2, SDHA, CS). Patient numbers for the genes (GOT1, ALDH7A1, LAP3, and ALDH9A1) was n = 1169, whereas for genes ACO2, IDH2, citrate synthase, SDHA, MPC1, and MPC2 it was n = 1150. Log ratios of the red and green processed signals (i.e., background-corrected and dye-normalized) were calculated, as well as a log ratio error and a *p*-value for each feature. For gene expression two-color arrays, these calculations assess the confidence that the gene is or is not differentially expressed. The expression of the latter in these datasets was visualized as a heat map using the z-scores of the expression in the individual patients. For the datasets GSE11318 and GSE87371 clinical-stage information was also available. The overall survival information for the GSE10846, GSE11318, GSE87371, and the progression-free survival for the GSE87371 and GSE93984 datasets were pooled and performed in GraphPad Prism 9.0.2 with a Kaplan–Meier method and log-rank p statistics. All the figures and statistics were conducted in GraphPad Prism 9.0.2. Analysis of responders and non-responders to Ibrutinib was assessed. The expression data of the publicly available dataset GSE93984 was downloaded from GEO.

### 2.13. Xenograft Study

Animal studies were completed under protocols for animal welfare approved by the Institutional Animal Care and Use Committee (2018A00000134, The Ohio State University, Columbus, OH, USA). Briefly, seven-week-old NSG mice were tail vein engrafted with 1 × 10^6^ of passage 1 DFBL-47933 cells from an ABC-DLBCL patient-derived xenograft (PDX) mouse model obtained from the public repository of xenografts (Proxe) [25]. Mice were then randomized to receive either vehicle control (n = 5) or ibrutinib 30 mg/kg in drinking water (n = 10) starting at day 57 post engraftment (disease detectable by flow cytometry in the peripheral blood). All animals were monitored daily for signs of tumor burden, including hind limb paralysis, respiratory distress, weight loss, ruffled coat, and distended abdomen, and euthanized when removal criteria were met. Body weight was measured weekly. The primary endpoint of these studies was survival defined as the time from engraftment to the development of the defined clinical criteria leading to the removal from the study. NSG mice were purchased from Jackson. 

## 3. Results

### 3.1. Ibrutinib-Resistant DLBCL Lines

**Figure 1:**
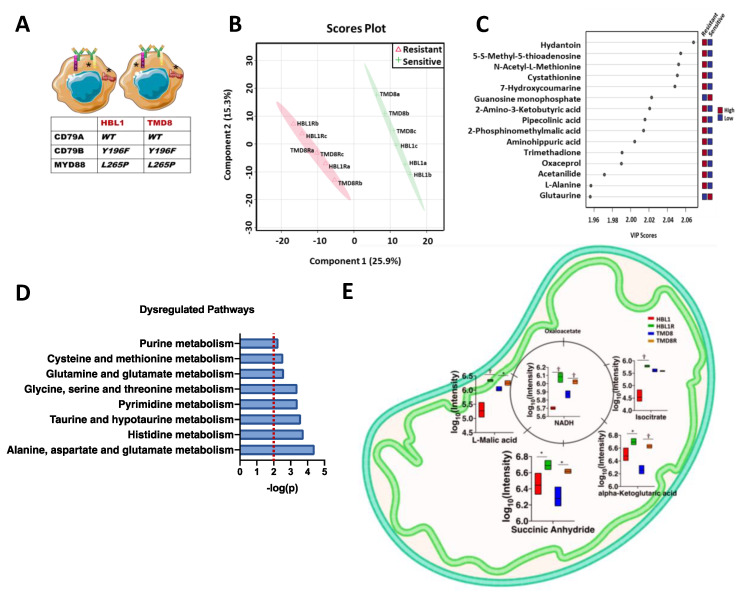
Metabolic profiling of the ibrutinib-resistant ABC-DLBCLs (HBL-1 and TMD-8). (**A**) Wild—type HBL1 and TMD8 cells were selected for their B cell receptor (BCR) independence and MYD88 gene expression (exhibiting mutation in CD79B (Y196F) and MYD88 (L265P). (**B**) PLS-DA plot depicting the variability in metabolic profiles of sensitive (HBL-1 and TMD-8) versus its resistant clone (HBL-1R and TMD-8R). (**C**) VIP plot showing the metabolites driving separation of the uniqueness in metabolic profiles. (**D**) Pathway analysis revealing the top significantly dysregulated metabolic pathways across the two phenotypes, based on −log(p) cutoff of 2 and pathway impact >0.2 depicted by dashed red line. (**E**) TCA cycle metabolites enrichment in sensitive vs resistant phenotype showing increased oxidative phosphorylation. The metabolites are represented as log_10_ intensity and unpaired students t-test was used for statistical analysis (*p* < 0.05).

Ibrutinib-resistant clones of the DLBCL cell lines HBL1 and TMD8 were generated by our group in vitro as reported previously [22] and named HBL1-R, TMD-8-R hereafter. Activated B-cell lymphoma pathogenesis exhibits irregular initiation of the BTK–mediated B-cell receptor signaling pathway [26] based on the genetic mutation in the BCR signaling pathways (Figure 1A). Ibrutinib, a selective and irreversible BTK inhibitor, has been successfully utilized in various leukemia and lymphoma models [27]. Along with resistant clones, wild-type cells were challenged with ibrutinib to confirm a shift in drug tolerance before experiments. Half-maximal inhibitory concentration (IC_50_) assays of the lymphomas cultured in incremental doses of ibrutinib confirm the acquired drug-resistance in these DLBCL lymphomas (Supplementary Figure S1A). BTK mutations (BTK-C481S) drive ibrutinib resistance and allow for continued BCR pathway signaling even in the absence of ibrutinib. In contrast to the BTK C481S mutation, PLCG2 gain-of-function mutations (PLCG2-R665W, PLCG2-S707Y, and PLCG2-L845F) allow for continued signaling regardless of BTK activity [28,29,30]. Targeted sequencing of both ibrutinib-resistant DLBCL cell lines did not identify these genetic alterations in these two genes as reported previously [22]. Ibrutinib resistance appeared to be accompanied by a more rapid doubling rate in the HBL1R and TMD8R cell lines than their ibrutinib-sensitive parental lines (Supplementary Figure S1B). Recently, we have shown that BCR signaling-dependent ABC-DLBCL cells lack B-cell signaling upon acquiring ibrutinib resistance [22]. We hypothesized that the acquired ibrutinib resistant ABC-DLBCL (HBL1-R-and TMD8-R) that lack BCR signaling is more likely to exhibit metabolic alteration.

### 3.2. Determining the Metabolic Modifications Accompanying the Ibrutinib Resistance Phenotype 

To further confirm if the acquired ibrutinib-resistant cells exhibited metabolic alterations, we performed a comprehensive untargeted metabolomics analysis with extensive compound identification processes using multiple databases, including the human metabolite database (HMDB), Kyoto encyclopedia of genes and genomes (KEGG), and PubChem compound database, and our in-house high-resolution mass spectra database. Our analysis identified 604 intracellular polar metabolites mutually detected in both HBL1, TMD8, and respective resistant clones. Partial least square discriminant analysis (PLS-DA) was performed to observe the metabolic differences of two cell phenotypes (Figure 1B). PLS-DA component 1 (*x*-axis) summarized 25.9% of the variance. In comparison, 15.3% of variations (*y*-axis) of our detected metabolites was explained by component 2. As demonstrated, the biological replicates of the two cell lines were tightly clustered within their groups, ensuring good biological reproducibility (Figure 1B). More importantly, the experimental groups were separated from each other, validating their distinct metabolic profile (Figure 1B). The top 15 metabolites driving separation of the clusters in the PLS-DA plot were identified and showed in the variance importance in the projection (VIP) plot (Figure 1C). A greater VIP score indicates the higher importance of these metabolites in driving the separation of the drug-resistant and drug-sensitive cell lines. The color map on the right of the VIP plot of Figure 1C shows the relative abundance of these metabolites. As these individual metabolites partake in complex metabolic processes, a summary of the major altered pathways was generated. The eight most impacted metabolic pathways were selected based on the impact score >0.2 and −log (p) > 2 (Figure 1D). At the crosstalk of various pathways reported in Figure 1D, 44 metabolites from the significantly deregulated pathways in resistant cells were detected. The results were compared to sensitive cells (Supplementary Figure S1C). To confirm if the deletion in the immunoreceptor tyrosine-based activation (ITAM) domain of CD79a [13] altered the metabolic profiling of ibrutinib-resistant clones, we performed unbiased metabolic integration on the previously characterized [22] OCI-LY10-R. Metabolite pathway analysis on OCI-LY10R yielded 14 most impacted metabolic pathways based on the impact score >0.2 and −log (p) > 2 (Supplementary Figure S1D). Further overlapping of the metabolic pathways from all the resistance cell types (HBL1R, TMD8R, and OCI-LY10R) yielded seven common pathways (Supplementary Figure S1E). Alanine, aspartate, and glutamate metabolism were revealed to be among the most deregulated pathways. Metabolites, including prominent pathway intermediates from cysteine and methionine metabolism (e.g., cystathionine), significantly drove the separation of the groups (Supplementary Figure S1E). As glutamate is readily converted to an alpha-ketoglutarate, a key metabolite that potentiates the tri-carboxylic acid cycle (TCA) [31], we investigated whether the levels of the other metabolites in TCA were also deregulated. As shown in Figure 1E, the major tricarboxylic acid (TCA) cycle metabolites such as L-malic acid, isocitrate, α-ketoglutarate, succinate anhydride, and their respective enzymes citrate synthase, aconitase, IDH2, and SDHA were also relatively more abundant in both HBL1-R and TMD8-R cells (Figure 1E and Supplementary Figure S2A), suggesting that ibrutinib-resistant cells shifted towards oxidative phosphorylation.

### 3.3. Discovering a Genetic Network Driving Altered Metabolic Pathways Using Multiomics Integration 

**Figure 2:**
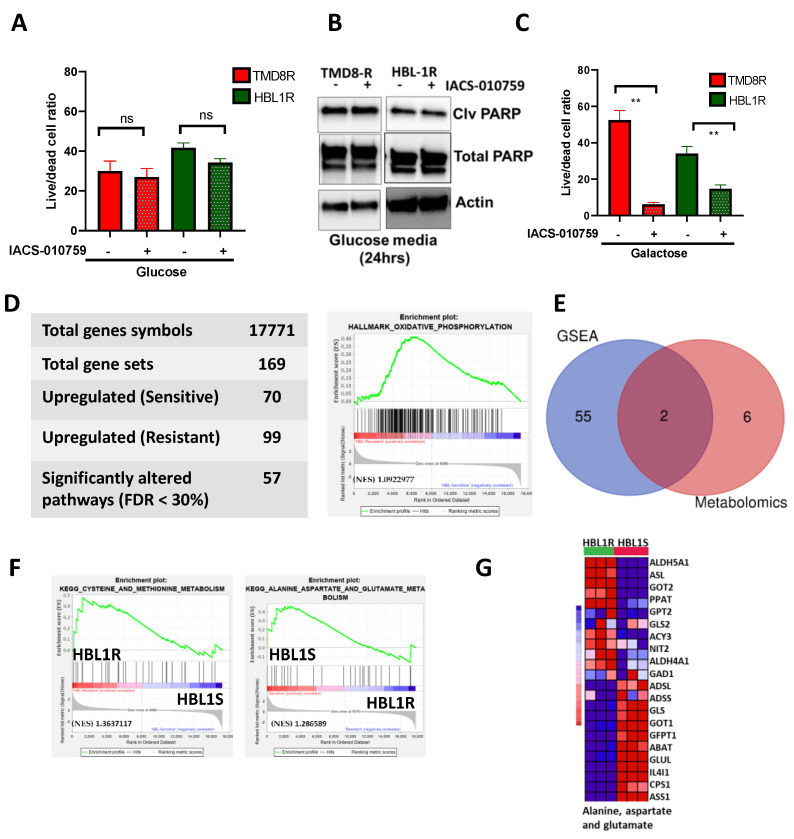
Ibrutinib-resistant ABC DLBCL clones favor oxidative phosphorylation. (**A**) Quantification of mean ratio ± sd of the percentage of live/dead cells at 24 h, extrapolated from the data acquired from glucose assay on ibrutinib-resistant clones in presence or absence of ETC chain inhibitor IACS-010759. Paired student t-test was used for statistical analysis (* *p* < 0.05, ** *p* < 0.001). (**B**) Western blot analysis of the cleaved PARP and total PARP in resistant cells cultured in glucose media in presence or absence of IACS-010759. (**C**) Quantification of mean ratio ± sd of the percentage of live/dead cells at 24 h, extrapolated from the data acquired from galactose assay on ibrutinib-resistant clones in presence or absence of ETC chain inhibitor IACS-010759. Paired student t-test was used for statistical analysis (* *p* < 0.05, ** *p* < 0.001). (**D**) Summary of the gene set enrichment analysis of HBL1/HBL1R expression data from the DNA microarray showing enrichment in oxidative phosphorylation. (**E**) Overlap of gene and metabolite data shows two altered metabolic pathways at both the metabolic and transcriptional levels (cysteine and methionine metabolism and alanine, aspartate and glutamate metabolism) and their respective GSEA enrichment plot. (**F**) Summary of the gene set enrichment analysis of HBL1/HBL1R expression data from the DNA microarray showing enrichment in cysteine and methionine and alanine, aspartate and glutamate metabolism. (**G**) Heatmap indicating the altered genes in cysteine and methionine as well as alanine aspartate and glutamate metabolic pathways.

Lymphomas are generally considered glycolytic [32]. As reprogramming of energy metabolism is a hallmark of cancer progression [33], we questioned whether a shift to oxidative phosphorylation (OXPHOS) occurred in the drug-resistant clone. Toward this end, we used galactose as an alternate carbon source in our culture medium. Compared to 10 mM glucose, ATP yield from 10 mM galactose was slower [34], and cancer cells used glycolysis to a lesser extent when galactose was provided as a sugar source. Thus, galactose and exogenous mitochondrial OXPHOS substrates led to a compensatory upregulation of OXPHOS [35]. As shown in Figure 2A,B, ibrutinib-resistant HBL1R and TMD8R cell survival remained unaffected when glucose was supplemented in the medium. IACS-010759 selectively inhibits complex I of the mitochondrial electron transport chain (ETC), thereby disrupting OXPHOS, the metabolic process some tumor cells rely on for growth and survival [36]. Further, the addition of ETC inhibitor IACS-010759 in glucose medium failed to induce further cell death in 24 h, as depicted by unchanged levels of the live/dead cell ratio and cleaved PARP (Figure 2A,B). Substitution of glucose with galactose medium in ibrutinib-resistant cells further sensitized them to ETC inhibitor IACS-010759 within 24 h (Figure 2C), suggesting that resistant cells had an active OXPHOS signaling, which IACS-010759 can target. While untargeted metabolomics provided a plethora of data, it represents a biological snapshot of the metabolic profiles of the cells at the time of harvest [37]. To further enable mechanistic understanding, transcriptomic data from the HBL1 and HBL1R cell lines were also generated to further validate the observed metabolic changes. As reported in Supplementary Figure S2D, gene set enrichment analysis (GSEA) of DNA microarray data was conducted to observe changes of genes along various metabolic pathways based on the curated KEGG gene set from the molecular signatures database. A total of 169 deregulated gene sets between the HBL1 and HBL1R were found, of which 99 were upregulated in the resistant cells. A false discovery rate (FDR) cutoff of 0.3 was used to increase confidence in the data, rendering 57 significant gene sets that may be significantly altered when HBL1 cells acquire drug resistance. GSEA analyses revealed high OXPHOS (Figure 2D) and PI3K-AKT MTOR signaling and MTORC1 signaling (Supplementary Figure S2B). Further combining the dataset from both HBL1R and OCI-LY10R also revealed the high association of OXPHOS in the ibrutinib-resistant cells (Supplementary Figure S2C), suggesting ibrutinib resistance metabolically favors oxidative phosphorylation. The overlap of the top 57 altered pathways from microarray GSEA along with the 8 deregulated pathways from untargeted metabolomics analysis highlighted that there were 2 significantly deregulated metabolic pathways at both the transcript and metabolite level (Figure 2E). The two detected pathways encompassed (1) cysteine and methionine metabolism, and (2) alanine, aspartate, and glutamate metabolism. Alanine, aspartate, and glutamate metabolism was down-regulated in the resistant phenotype; conversely, cysteine and methionine metabolism was found to be upregulated (Figure 2F). These results suggest that resistant cells are metabolically reprogramed to utilize oxidative phosphorylation as a source of energy.

### 3.4. Multi-Omics Integration Highlights Metabolic Shift towards Oxidative Phosphorylation with IL4I1 at the Intersection 

**Figure 3:**
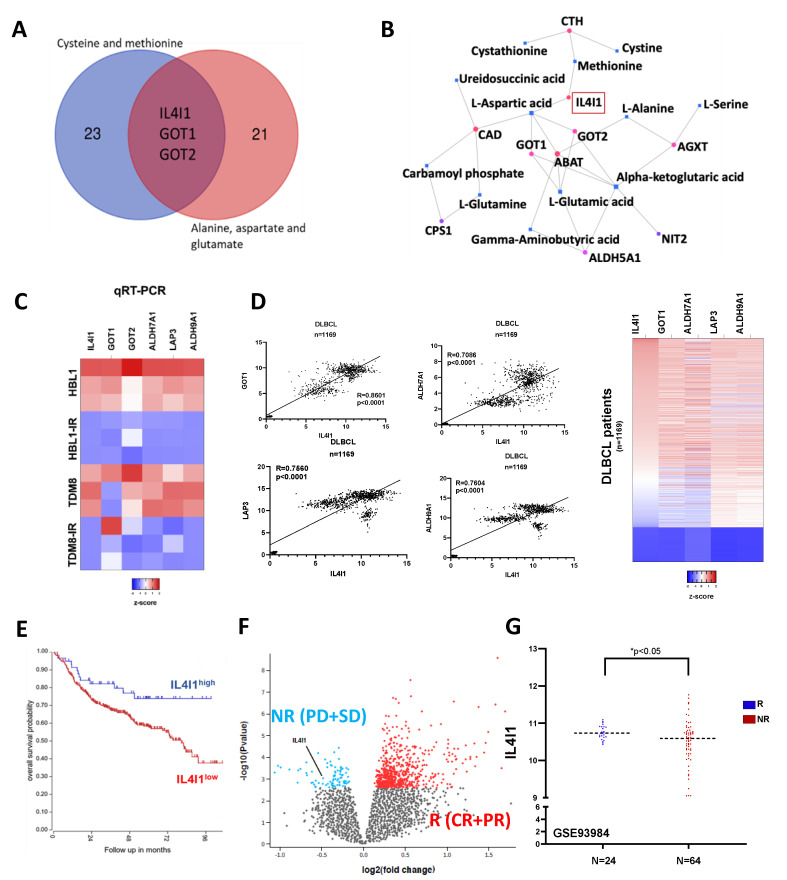
Integration of metabolic genes highlight IL4I1. (**A**) Overlap of the genes pertaining to each metabolic pathway revealed IL4I1, GOT1, and GOT2 to be at the crosstalk of these pathways. (**B**) Proposed interaction map of transcriptomic and metabolomic data, regulated by IL4I1. (**C**) q-PCR quantification of the genes of interest in lymphomas to validate expression in ABC-DLBCL ibrutinib-resistant cells. (**D**) Correlation plot and heatmap of the IL4I1 with the key metabolic genes in the 1169 DLBCL patients. (**E**) Kaplan-Meier survival analysis of DLBCL patients as determined from GSE31312 and analyzed by R2: Genomics Analysis and Visualization Platform. (**F**) Volcano showing the IL4I1 low levels in ABC DLBCL patients who did not respond to ibrutinib, as determined from the GSE93984. (**G**) Relative level of IL4I1 in ABC DLBCL patients who did not respond to ibrutinib.

We identified genes overlapping in both deregulated pathways to further investigate these metabolic changes and confirm the observed trend in ibrutinib-resistant DLBCL metabolism (Figure 3A). The enzymes interleukin 4 induced 1 (IL4I1) and glutamic-oxaloacetic transaminase 1 and 2 (GOT1 and GOT2) were implicated in both sets of metabolic pathways. IL4I1 belongs to the L-amino-acid oxidase family and catalyzes the oxidation of L-phenylalanine to keto-phenylpyruvate [38]. The IL4I1 protein also works in conjunction with additional amino-transferases to target other amino acids. Gene-metabolite interactions from our two deregulated pathways were analyzed and mapped (Figure 3B). This interaction network revealed that IL4I1 links methionine and aspartic acid, thereby bridging the gap between cysteine and methionine metabolism and alanine, aspartate, and glutamate metabolism. These proteins mediate the reversible transamination from glutamate to oxaloacetate, generating α-ketoglutarate and aspartate [39]. As shown in Figure 3C, IL4I1 mRNA expression was analyzed by q-PCR, represented in the heatmap, and found at significantly decreased levels in HBL1R (*p*-value = 0.014) TMD8R (*p*-value = 0.0052) compared to wild-type HBL1 and TMD8, respectively. GOT1 and GOT2 mRNA expression levels were significantly decreased in HBL1R (*p*-value = 0.013) and TMD8R (*p*-value = 0.02), respectively. Additionally, gene levels of drug resistance targets ALDH7A1, ALDH9A1, and LAP3 were diminished in both resistant cells. To confirm the rigor and reproducibility of our data, we queried GSE93985 and determined the levels of IL4I1 and other key genes (Supplementary Figure S2D). Consistent with our data, levels of IL4I1 and other key genes were down-regulated in the dataset GSE93985. Further, the IL4I1 expression levels correlated positively with GOT1, LAP3, ALDH7A1, and ALDH9A1 in 1169 DLBCL patients (Figure 3D), suggesting that IL4I1 positively regulates key genes known to regulate drug resistance. Kaplan–Meier survival plots were generated for DLBCL patients using the publicly accessible R2 genomics visualization platform [40]. Gene Expression Omnibus data repository number GSE31312 was used to analyze IL4I1 levels to determine if there was a relationship between disease outcome and gene expression [41,42]. Decreased expression of IL4I1 was directly correlated with lower chances of survival (*p*-value = 0.019) (Figure 3E). Further analysis of GSE93984 (Figure 3F,G) showed that IL4I1 levels were low in non-responders (n = 64) that received ibrutinib phase II PCYC-1106 trial (NCT01325701) [26], suggesting IL4I1 can modulate ibrutinib resistance in ABC-DLBCL.

### 3.5. Wild-Type BTK Expression Restored IL4I1 in Resistant Tumors Contributes to Resistance and Metabolic Reversal

**Figure 4:**
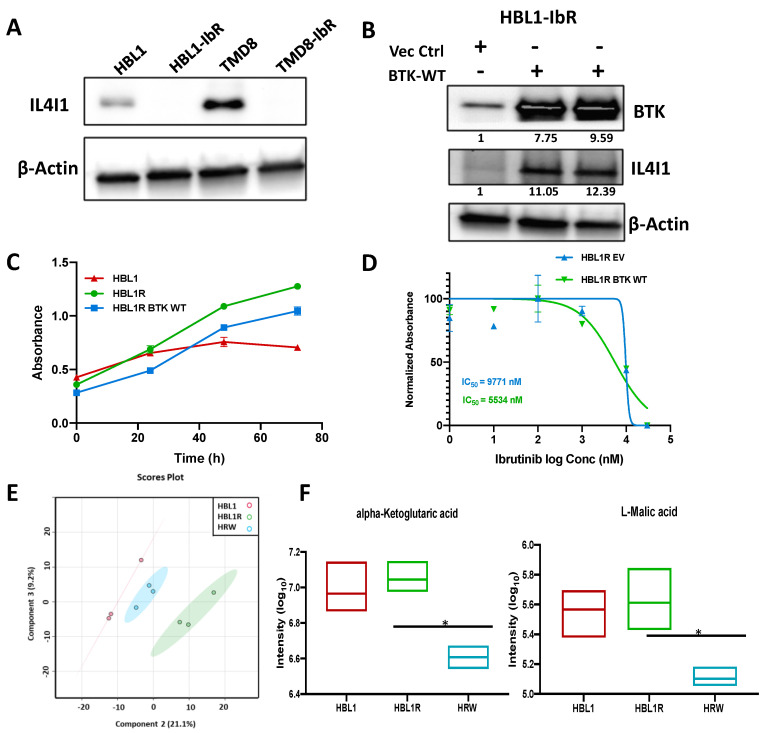
Wild-type BTK expression in the drug-resistant cells hints at the partial metabolic reversal. (**A**) Western blot of baseline IL4I1 expression across the lymphomas and their resistant clones. (**B**) Western blot of IL4I1 expression upon ectopic expression of BTK WT in ibrutinib-resistant HBL1 cells (values below blots represent relative densitometry quantification value compared to vector control). (**C**) MTT proliferation assay to determine cell growth in the WT-BTK-expressing resistant cells compared to their respective parental line. (**D**) Representative IC50 graphs of the WT-BTK expressing HBL1 ibrutinib—resistant lymphomas treated with ibrutinib. (Please note that the ectopic expression of the empty vector alters the basal IC50 in resistant clones.) (**E**) PLS-DA plot depicting the variability in metabolic profiles of HBL1, HBL1R, and HBL1R expressing WT—BTK (HRW). (**F**) Relative signal intensity of TCA metabolites (alpha-ketoglutaric acid and malic acid) across the HBL1 derived cells. * *p* < 0.05.

The level of IL4I1 is downregulated in the ibrutinib-resistant clones in both cell types at protein (Figure 4A) and mRNA level (Figure 3C). To test whether the loss of BTK changed the levels of IL4I1 and affected ibrutinib resistance, we ectopically expressed the wild-type (WT) BTK in ibrutinib-resistant cells and measured the expression of IL4I1. Compared to controls, IL4I1 levels increased upon ectopic expression of either WT-BTK in HBL1-R (Figure 4B) and TMD8-R (Supplementary Figure S3A). Ectopic expression of wild-type BTK delayed cell proliferation (Figure 4C and Supplementary Figure S3B). Wild-type BTK increased the sensitivity of the HBL1R-derived cells to ibrutinib (Figure 4D) and marginal change in the TMD8R-derived cells (Supplementary Figure S3C). Densitometry quantification showed that the ectopic expression of BTK in TMD8R was nearly half as compared to HBL1R, with respect to ibrutinib resistance, since the ectopic expression of the BTK in TMD8R was marginal, and more BTK expression levels are required in TMD8R to achieve more sensitization to ibrutinib. These results suggest that the expression of BTK can regulate the level of IL4I1 and may contribute to ibrutinib resistance. Our previous analysis showed that loss of BTK activates the PI3K-AKT pathway. To test whether the PI3K-AKT axis regulates IL4I1 expression, we treated the cells with BEZ235, a dual inhibitor of mTOR and AKT. As shown in Supplementary Figure S3D, the IL4I1 level was restored after treating with the dual inhibitor, suggesting that IL4I1 is partly regulated by PI3K-AKT signaling. To further ascertain whether the restored IL4I1 levels in HBL1R-WT-BTK affected the metabolism of the drug-resistant clone, untargeted metabolomics were performed on the clone. Our analysis revealed 458 intracellular polar metabolites mutually detected in the transduced mutants and annotated using the previously described spectral databases. To observe the metabolic similarities between the two cell phenotypes, PLS-DA was performed. PLS-DA plots revealed that the wild-type BTK expression group clustered adjacently to the wild-type HBL1 groups rather than HBL1R, suggesting a more similar metabolic profile to sensitive cells (Figure 4E). We found that the levels of crucial metabolites were reversed, such as α-ketoglutarate (*p*-value = 0.017), suggesting that the expression of BTK can reverse the expression of IL4I1 and the associated metabolic changes (Figure 4F). Similarly, TMD8R cells transduced with wild-type BTK (TRW) clustered further away from the parental TM8R cells and closer to the sensitive cells, suggesting a more similar metabolic pattern to TMD8 (Supplementary Figure S4A). In addition, BTK can reverse the expression of IL4I1 and the associated metabolic changes in TMD8-derived cells (Supplementary Figures S3A and S4B). IACS-010759 selectively inhibits complex I of the mitochondrial electron transport chain (ETC), thereby disrupting OXPHOS, the metabolic process some tumor cells rely on for growth and survival [36]. Further, we found that the ibrutinib-resistant cells HBL1R were now more sensitive to the treatment by IACS-010759 (Supplementary Figure S4C), suggesting that metabolic reprogramming toward oxidative phosphorylation can be targeted therapeutically using OXPHOS inhibitors.

### 3.6. Loss of IL4I1 in Cells, DLBCL Patients, and PDX Mouse Contributes to Resistance and Metabolic Reversal

**Figure 5:**
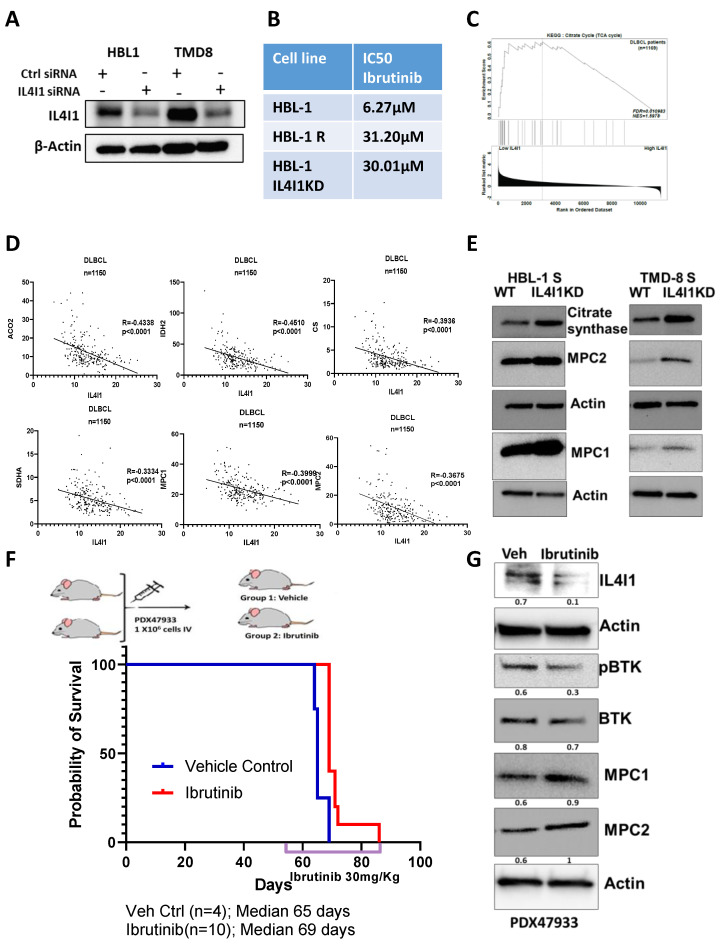
Loss of IL4I1 correlates with TCA cycle enzymes and mimics resistance to ibrutinib. (**A**) Western blot of baseline IL4I1 expression upon transfection by siRNA specific to IL4I1 or scrambled siRNA in HBL1 and TMD8 cells. (**B**) Table depicting IC50 concentrations of the HBL1 cells with either control or IL4I1 siRNA or HBL1-resistant lymphomas treated with ibrutinib (please note that the genetic manipulation alters the basal IC50 in resistant clones). (**C**) Low IL4I1 levels in ibrutinib-resistant ABC—DLBCL patients are enriched for TCA cycle enzymes as determined by GSEA on GSE93984. (**D**) Correlation plot of the IL4I1 with 1150 DLBCL patients. (**E**) Western blot analysis of TCA enzymes in HBL1 and TMD8 ibrutinib-sensitive cells transfected with IL4I1siRNA. (**F**) Kaplan–Meier curve demonstrating the survival of ibrutinib-treated ABC- DLBCL PDX in NSG mice. (**G**) Western blot analyses on isolated pooled splenocytes from the vehicle control or ibrutinib-treated ABC-DLBCL patient-derived xenograft and probed for the labeled antibodies (values below blots represent relative densitometry quantification value compared to actin).

To further confirm if IL4I1 can contribute to drug resistance, we knocked down IL4I1 in HBL1 and TMD8 parental cells at the protein level using specific small interfering RNA (siRNA) targeting IL4I1, and scrambled siRNA served as control (Figure 5A). Further, the loss of IL4I1 in the parental cells elicited ibrutinib resistance similar to the acquired resistant clones (Figure 5B and Supplementary Figure S5A). GSEA analysis on IL4I1 low vs. high patient GSE93984 (Figure 5C) confirmed that the loss of IL4I1 in DLBCL patients who received ibrutinib correlated with upregulation of TCA cycle enzymes. We found that DLBCL patients that expressed low levels of IL4I1 had enrichment of TCA cycle enzymes. This result was further strengthened when we discovered that IL4I1 negatively correlated with TCA cycle enzymes (ACO2, IDH2, CS, SDHA, MPC1, and MPC2) in 1150 DLBCL patients (Figure 5D). Further by targeting IL4I1 using specific siRNA in HBL-1 and TMD-8 sensitive cells, we found elevated TCA cycle enzymes such as citrate synthase, MPC2, and MPC1 (Figure 5E). These results suggest that loss of IL4I1 in ibrutinib-sensitive cells upregulates the TCA cycle enzyme. One of the critical challenges well known in the field is the limitation of the patient tissue biopsy for establishing an ibrutinib-resistant ABC DLBCL with MYD88 and CD79A and B mutations. Alternatively, we utilized a patient-derived xenograft mouse model from an ABC-DLBCL patient received from the public repository of xenografts (PROXE). This experiment aimed not to generate an ibrutinib-resistant model, but instead to question if ibrutinib treatment in vivo mimicked our in vitro results. Mice were then randomized to receive either vehicle control (n = 5) or ibrutinib 30 mg/kg in drinking water (n = 10) starting at day 57 post engraftment (disease detectable by flow cytometry in the peripheral blood) (Figure 5F). To further show if ibrutinib treatment conferred loss of IL4I1 levels and negatively correlated with TCA enzymes in vivo, we isolated the pooled splenocytes from patient-derived xenograft PDX47933 and blotted for IL4I1, BTK, and TCA enzymes (Figure 5G). We found that loss of IL4I1 in ibrutinib-treated PDX led to an increased level of TCA enzymes, similar to our results in Figure 5E, suggesting that IL4I1 loss can modulate ibrutinib resistance and trigger metabolic shift to OXPHOS. 

### 3.7. The Proposed Intersection of Amino Acid Metabolism and TCA Cycles within the Ibrutinib Resistance Mechanism Regulated by IL4I1

**Figure 6:**
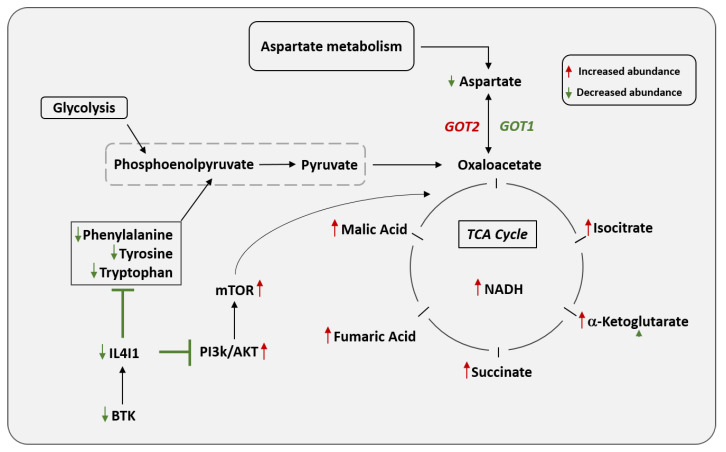
Proposed crosstalk of amino acid metabolism and TCA cycles within the ibrutinib resistance mechanism regulated by IL4I1. The proposed metabolic cross-talks in relation to TCA anaplerosis in drug-resistant lymphoma. Modulation of BTK and PI3K AKT pathways regulates the expression of IL4I1. Decreased IL4I1 expression affects phenylalanine metabolism, accompanied by decreased pyruvate levels, shifting to increased oxidative phosphorylation, and driving robust cell proliferation in drug-resistant lymphoma. One cannot exclude the possibility that ancillary metabolic pathways, such as the urea cycle as well as glutamate and glutamine metabolism, can directly contribute to TCA intermediates fumaric acid and α-ketoglutarate, respectively.

As resistant lymphoma metabolism is established on an intersection of amino acid metabolic pathways, we continued our effort to show a gene–metabolite network to identify a mechanism for the observed replenishing of the TCA cycle. Although it is still unclear how IL4I1 can regulate metabolism, we proposed a data-driven network to explain this TCA anaplerosis. Mapping genes constructed potential routes linking these metabolic processes to metabolites through the biochemical pathways to which they pertain. As shown in Figure 6, the TCA cycle was centered with several key metabolites detected in our study. These metabolites focused on opening a door for potential direct metabolic links to occur. Highlighted on the left of Figure 6, IL4I1 is under the regulation of BTK. Our data suggest that decreased expression of IL4I1 will halt numerous amino acids from being converted to acetyl-CoA, the main product of glycolysis. This allows a link for amino acids, such as alanine, to be converted to pyruvate to sustain energy metabolism. This provides a possible mechanism by which cells can propagate the TCA cycle in the absence of IL4I1.

## 4. Discussion

Our study showed that drug-resistant lymphoma lines favor OXPHOS in addition to glycolysis for energy production. Many types of cancer cells exhibit pronounced metabolic reprogramming compared to non-transformed cells, critical to oncogenesis. Oncogenic mutations at the core of carcinogenesis drive large-scale metabolic alterations, which are both native to cancers and obligatory for the transition to malignancy. The most notable of these metabolic alterations in the activation of aerobic glycolysis is termed the Warburg effect [43,44]. Other metabolic modifications that drive increased malignancy include activation of fatty acid biosynthesis and glutamine consumption [45]. Lymphoma metabolism has been extensively studied to identify metabolic perturbations across lymphoma subtypes [46]. DLBCL cells have been shown to metabolize more pyruvic acid, which is indicative of a glycolytic state. This finding was corroborated when levels of the glycolytic enzymes hexokinase 1 (HXK1) and phosphoglycerate kinase 1 (PGK1) were both determined to be upregulated in this lymphoma subset. Pathway analysis revealed increased expression of genes involved in glycine, serine, threonine, and pyrimidine metabolism in DLBCL. This is in line with identifying unique metabolic profiles of lymphoma patient plasma samples from each cancer subtype [47]. Compared to other lymphomas, DLBCL patients were found to have increased circulating levels of glutamic acid and glycine, coupled with decreased levels of aspartic acid, tryptophan, and uric acid. These reports are not fully mirrored in our data, suggesting a unique metabolic profile of the resistant cells.

By integrating the metabolomics data with transcriptomic data, we uncovered an amino acid oxidase, IL4I1, which mediates TCA anaplerosis in ibrutinib-resistant DLBCL (Figure 6). Potential cross-talk that may justify TCA anaplerosis is that of the urea cycles. These two cycles are closely linked, providing a means for substrates to transition between both pathways. This link is denoted by the aspartate-argininosuccinate shunt (Supplementary Figure S5C), which effectively connects the fates of the amino groups and the carbon skeletons of amino acids [48]. In normal tissues, urea cycle proteins are the only providers of endogenous metabolites (such as arginine, ornithine, and citrulline) that are required by other metabolic pathways to sustain function [49]. Deregulated expression of urea cycle intermediates is linked to decreases in nitrogen waste production and increased redirection of carbon and nitrogen carriers to biosynthetic processes [50]. As various enzymes in the urea cycle can potentially redirect metabolites to support cancer progression, numerous cross-talks exist between the urea cycle and other metabolic pathways. Two urea cycle enzymes, argininosuccinate synthase (ASS1) and argininosuccinate lyase (ASL), were detected in our study and are involved in the metabolism of citrulline and arginine (Figure 2G). Their enzymatic activities provide amino acids to be used as substrates for nitric oxide, proline, and other substrates needed by the cell for sustained survival and growth [51]. Oxaloacetate from the TCA cycle can be converted to aspartate, which can be acted upon in turn by ASS1 to produce the urea cycle metabolite, argininosuccinate. This newly formed aspartate can present a nitrogen entry point to the urea cycle. Argininosuccinate can be converted to fumaric acid via ASL, suggesting anaplerosis of the citric acid cycle. TCA anaplerosis was evident by the increased expression of ASL (Figure 2G) coupled with an increased abundance of fumaric acid. However, as argininosuccinate and ASS1 levels were lowered in the phenotypically resistant cells (Figure 2G), we explored other TCA links. Ornithine from the urea cycle can be used for anabolic processes to synthesize metabolites that participate in the TCA cross-talk. Ornithine, whose levels were almost tripled in HBL1R compared to HBL1 (*p*-value = 0.009), can be combined with carbamoyl phosphate to form citrulline needed for argininosuccinate production by ASS1. Endogenous formation of carbamoyl phosphate occurs via carbamoyl phosphate synthetase (CPS1) when increased ammonia levels need to be condensed, and thus commences the urea cycle [52]. However, ASS1 and CPS1 expression levels were significantly downregulated in the resistant phenotype microarray; thus, this phenomenon may be altered to overcome drug treatment. Therefore, reduced gene expression hints at the possibility of alternative crosstalk.

Previous research has mainly highlighted the association of IL4I1 with the regulation of immune functions, with a reliance on amino acid depletion and the formation of H2O2 and toxic phenyl-pyruvic acid [53]. BCR-induced signaling is limited by IL4I1, consequently limiting B-cell proliferation. In mouse models, in vivo experiments reveal enhanced egress of naive B cells from the bone marrow in mice with IL4I1 knockout [54]. Loss of IL4I1 in resistant ABC-DLBCL shows lower pSHP1 (S591) (Supplementary Figure S2E) levels, which is consistent with previously published results in IL4I1 KO mice [54]. The IL4I1 protein participates in the metabolisms of numerous amino acids, including tryptophan, tyrosine, leucine, and valine [55]. However, not all of these metabolisms were significantly deregulated in the microarray data. By connecting the alanine, aspartate, and glutamate metabolism to that of cysteine and methionine, IL4I1 forms a direct link for these two metabolic pathways to communicate. It is known that aminotransferases can facilitate amino acid-mediated TCA anaplerosis; therefore, they are considered emerging determinants of oncogenesis [46]. GOT1 and GOT2 are the respective cytoplasmic and mitochondrial varieties of glutamic-oxaloacetic transaminase enzymes (Figure 6). They participate similarly to aspartate aminotransferases (AST) by catalyzing the reversible transamination of oxaloacetate and glutamate, producing aspartate and α-ketoglutarate [39]. The mitochondrial form of glutamic-oxaloacetic transaminase, GOT2, is a prominent player in the urea cycle and TCA cycle, particularly in the malate-aspartate shuttle [56]. The drug-resistant lymphoma lines reported in this study have been shown to favor OXPHOS for energy production, so a reduction in IL4I1 expression may hold the key to this metabolic adaptation. GOT1 was found to be significantly downregulated in the HBL1-resistant cells (Figure 3C). In TMD8R cells, inner membrane mitochondrial GOT2 was significantly downregulated (Figure 3C), suggesting decreased transamination of aspartate to form the TCA intermediate oxaloacetate (Figure 6 and Supplementary Figure S5C). Thus, these drug-resistant lymphomas need to recycle amino acid carbons to maintain energy production. Interestingly, studies have shown that the electron transport chain inhibition in cells lacking GOT1 reduces cell growth, thus providing evidence for the reliance of these cancers on OXPHOS metabolism [20,57]. As such, IL4I1 cannot serve its function as an L-amino acid oxidase, allowing excess phenylalanine to be converted to tyrosine. Tyrosine serves as an intermediate between phenylpyruvate and phenylalanine, and its reaction produces glutamate as a byproduct. With this, the cell has numerous recycling reactions that can generate glutamate for glutaminolysis. Glutamine and oxaloacetate can be respectively converted to α-ketoglutarate and aspartic acid via aspartate aminotransferase, which provides the cell a way to modulate the flux of pathways as needed [58]. It is believed that this provides a link between glycolysis, glutaminolysis, and TCA anaplerosis [45]. Conversion of glutamic acid to glutamine has been shown to fuel de novo purine biosynthesis [59]. As such, pyrimidine metabolism and purine metabolism were among the deregulated metabolic pathways indicating a direct effect on DNA and RNA synthesis. Our previous published study [22] suggests that loss of BTK can activate the PI3K AKT pathways. Here we show that activation of PI3K AKT pathway downregulates IL4I1 responsible for the metabolic reprogramming and drug resistance. We mapped out links that connect gene expression changes and metabolite changes to the ibrutinib-resistant phenotype. Whether a negative feedback loop exists between AKT and IL4I1 is not clearly understood; however, targeting this axis using a specific inhibitor of oxidative phosphorylation or PI3K-AKT pathway may hold the key to reverse and target the drug-induced metabolic adaptation. Our findings suggest that loss of IL4I1 induces a metabolic shift towards oxidative phosphorylation, a shared feature between the two DLBCL drug-resistant cell lines. Further, our integrative analysis highlighted the PI3K-AKT-IL4I1 axis in the drug-resistant lymphoma lines that favor OXPHOS for energy production. Targeting this axis using a specific inhibitor of oxidative phosphorylation or PI3K-AKT pathway may hold the key to reverse and target the drug-induced metabolic adaptation.

## 5. Conclusions

In this study, ibrutinib resistance was achieved in the DLBCL cell lines by perpetual culture in the presence of a BTK inhibitor. Cells exhibited an increased proliferation rate after overcoming the ibrutinib dose. IC50 and growth rate experiments confirmed this observation. The data strongly suggested that perturbations of lymphoma metabolism can be detected. A total of 167 gene sets and 603 metabolites were identified in the analysis of HBL1 and HBL1R, and their functional interactions were mapped. Significantly deregulated pathways from both datasets were overlapped, revealing mutually significant, reprogrammed metabolic pathways associated with drug resistance. Validation of the altered mechanisms was achieved using another DLBCL cell line, TMD8, and its resistant clone (TMD8R). Alanine, aspartate, glutamate, cysteine, and methionine metabolisms were deregulated in the resistant phenotype at both the metabolic and transcriptional levels. From the overlay of the individual pathway genes, we found the L-phenylalanine oxidase IL4I1 to be at the junction of both pathways. Further, our integrative analysis approach identified the PI3K-AKT-IL4I1 axis in the drug-resistant lymphoma lines that favor OXPHOS for energy production. This axis has been validated in independent datasets in vitro and in vivo using PDX xenograft model. Aberrant expression of the identified genes is accompanied by a shift towards oxidative phosphorylation in these cancers, which justifies the observed TCA anaplerosis and provides a mechanism of increased metabolic activity for survival. Targeting this axis using a specific inhibitor of oxidative phosphorylation or PI3K-AKT pathway may hold the key to reverse and target the drug-induced metabolic adaptation.

## Data Availability

The gene expression dataset is deposited in the Gene Expression Omnibus data repository under accession number GSE138126. The mass spectrometry raw data are available at MassIVE under accession number MSV000087059. All cell lines used and generated will be available upon request.

## Supplementary Materials

The following are available online at https://www.mdpi.com/article/10.3390/cancers13092146/s1, Figure S1. Characteristics of the ABC Ibrutinib-resistant DLBCL cells, Figure S2. Ibrutinib resistant ABC DLBCL clones favors oxidative phosphorylation, Figure S3. BTK Rescue experiments altered IL4I1 expression, Figure S4. Partial reversal in TMD8R cells upon overexpression of WT BTK gene, Figure S5. Aspartate-Arginosuccinate shunt of TCA cycle, Figure S6. Raw Blots for all the western blots for Figure 2B and SF2A, Figure S7. Raw Blots for all the western blots for SF2E, Figure 4A,B, SF3A, Figure S8. Raw Blots for all the western blots for Figure 5A,E,G.
