# Supplementary material for: Integration of Metabolomics and Gene Expression Profiling Elucidates IL4I1 as Modulator of Ibrutinib Resistance in ABC-Diffuse Large B Cell Lymphoma
Source: Cancers (Basel). 2021 Apr 29;13(9):2146. doi: 10.3390/cancers13092146 (PMC8124963; doi:10.3390/cancers13092146)
Supplement: Supplementary file 1 [file cancers-13-02146-s001.zip › cancers-1165562-supplementary.pdf]

cancers

MDPI

Integration of Metabolomics and Gene Expression Profiling Elucidates IL4I1 as Modulator of Ibrutinib Resistance in ABC-Diffuse Large B cell Lymphoma

Fouad Choueiry [1,#], Satishkumar Singh [2,3,#], Anuvrat Sircar [2,3], Georgios Laliotis [3,4], Xiaowei Sun [1], Evangelia Chavdoula [3,4], Shiqi Zhang [1], JoBeth Helmig-Mason [2], Amber Hart [2], Narendranath Epperla [2,3], Philip Tsichlis [3,4], Robert Baiocchi [2,3], Lapo Alinari [2,3], Jiangjiang Zhu [1,3,*] and Lalit Sehgal [2,3,*]

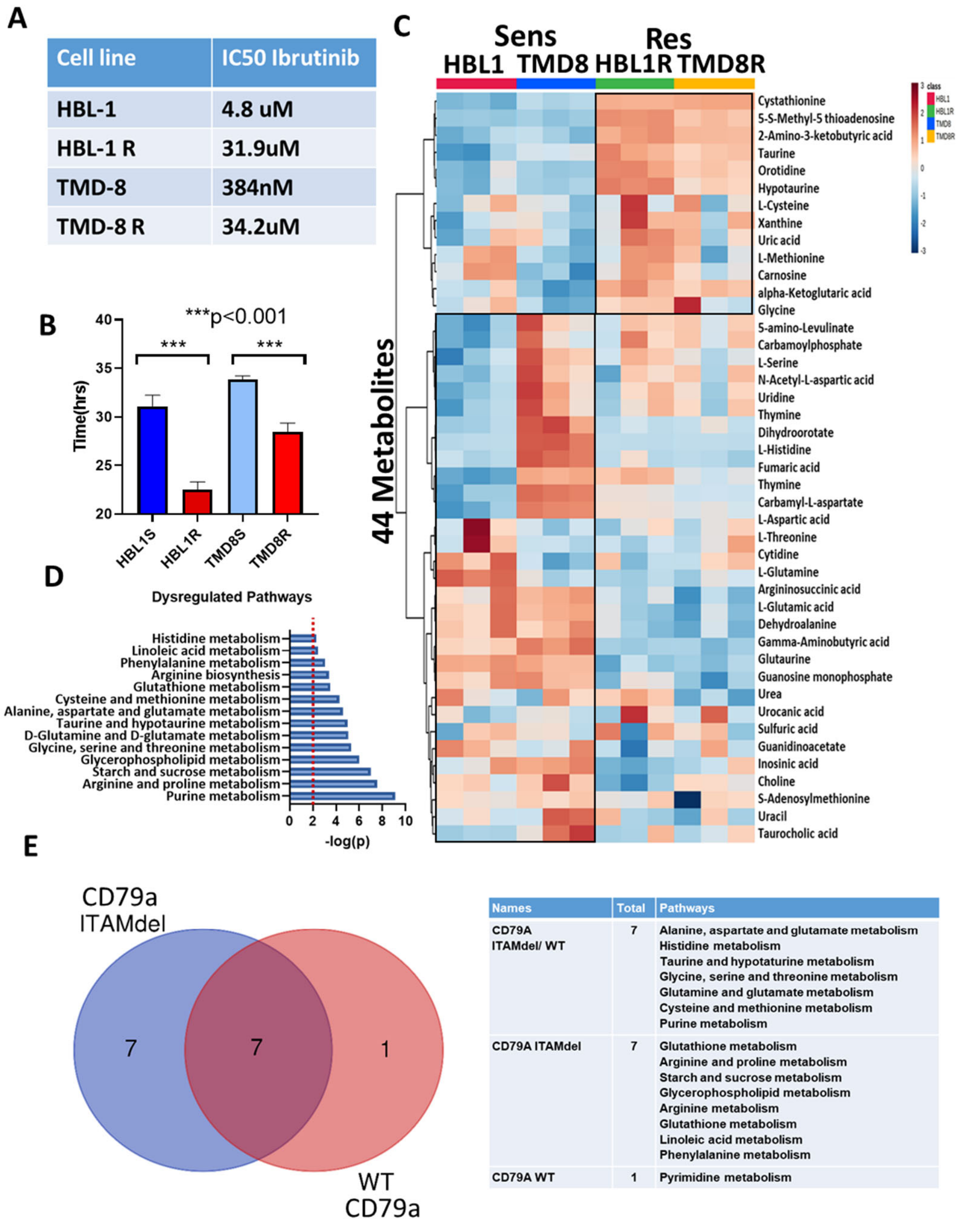

Figure S1. Characteristics of the ABC Ibrutinib-resistant DLBCL cells. A) Table depicting the Half-maximal inhibitory concentration (IC_{50}) of the lymphomas cultured in incremental doses of Ibrutinib. B) Doubling time for the HBL1R and

TMD8R compared to their respective wild-type parental lines. C) Heatmap of metabolites from significantly altered pathways revealing changes in levels among groups. D) Pathways analysis revealing the top significantly dysregulated metabolic pathways across OCILY10 and OCILY10R, based on –log (p) cutoff of 2 and pathway impact > 0.2 depicted by dashed red line. E) Venn diagram depicting the overlap metabolite data analyzed using CD79A-ITAM del cells OCI-LY10 and CD79A- WT cells (HBL1R/TMDR) shows seven common altered metabolic.

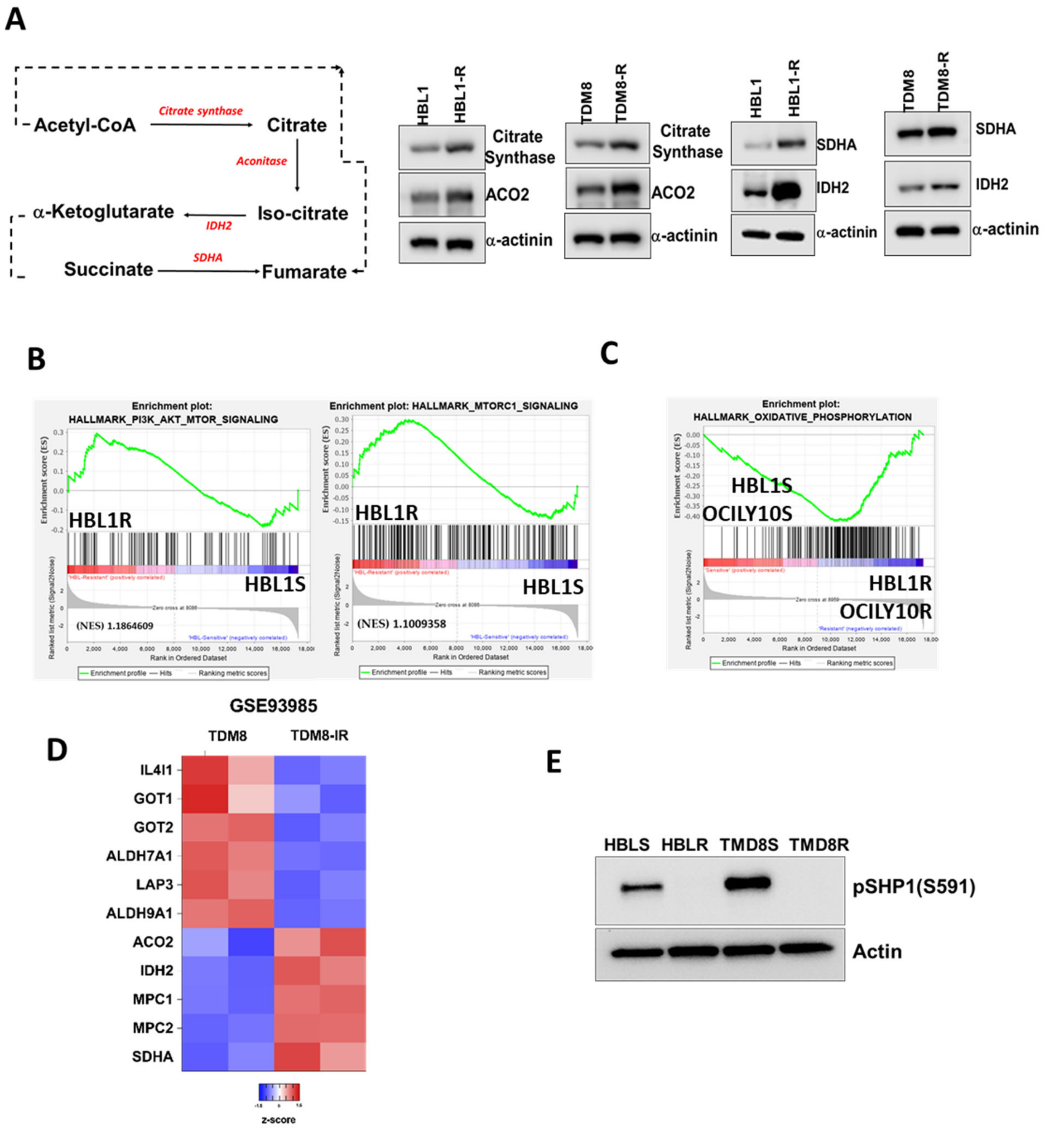

Figure S2. Ibrutinib resistant ABC DLBCL clones favors oxidative phosphorylation. A) Western blot analysis of the key regulatory enzymes of TCA cycle B-C) Summary of the gene set enrichment analysis of HBL1/HBL1R expression data from the DNA microarray showing enrichment in PI3K AKT MTOR signaling and MTORC1 signaling or oxidative phosphorylation (OXPHOS) when combined GSEA for HBL1R and OCILY10R was analyzed. D) Heatmap of the IL4I1 with the key metabolic genes in the TMD8 sensitive and TMD8R. E) Western blot analysis of the pSHP1 (S591) sensitive vs ibrutinib resistance ABC-DLBCL cells.

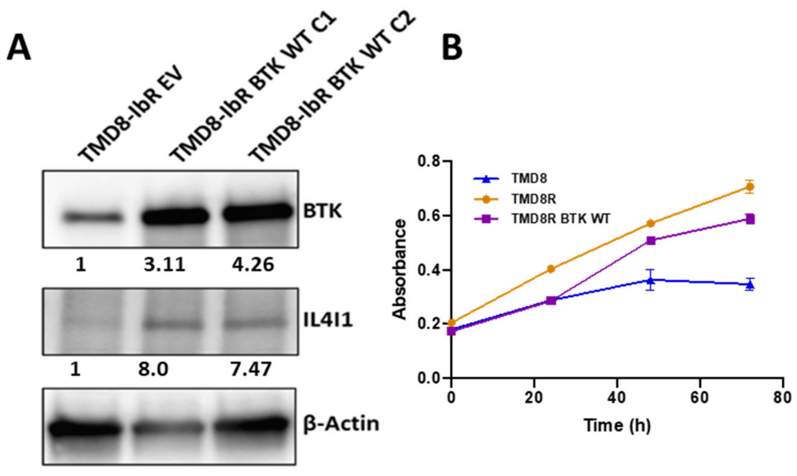

C

Cell line	IC50 Ibrutinib
TMD8R	10.11µM
TMD8R BTK WT	9.8 µM

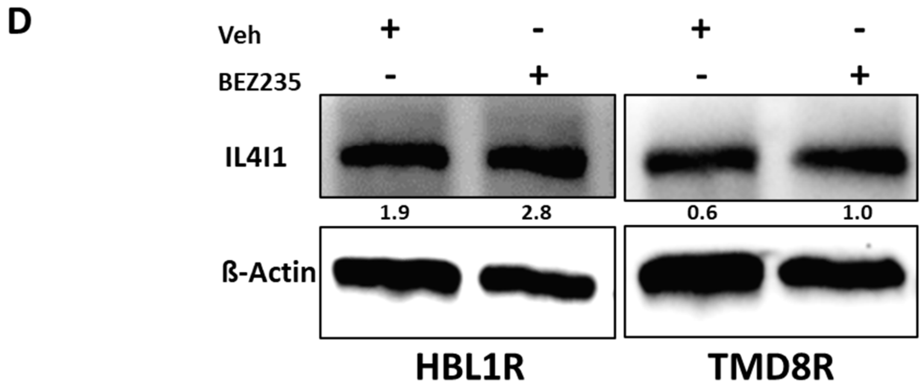

Figure S3. BTK Rescue experiments altered IL4I1 expression. A) Western blot of IL4I1 expression upon ectopic expression of BTK WT in ibrutinib resistant TMD8 cells (values below blots represent relative densitometry quantification value compared to vector control line). B) Proliferation assay to determine cell growth in the WT-BTK expressing resistant cells compared to their respective parental line. C) Table representing IC50 of the WT-BTK expressing resistant lymphomas treated with Ibrutinib. D) Western blot of IL4I1 expression in HBL1R and TMD8R upon treatment with PI3K/mTOR inhibitor (BEZ235) (values below blots represent relative densitometry quantification value compared to actin).

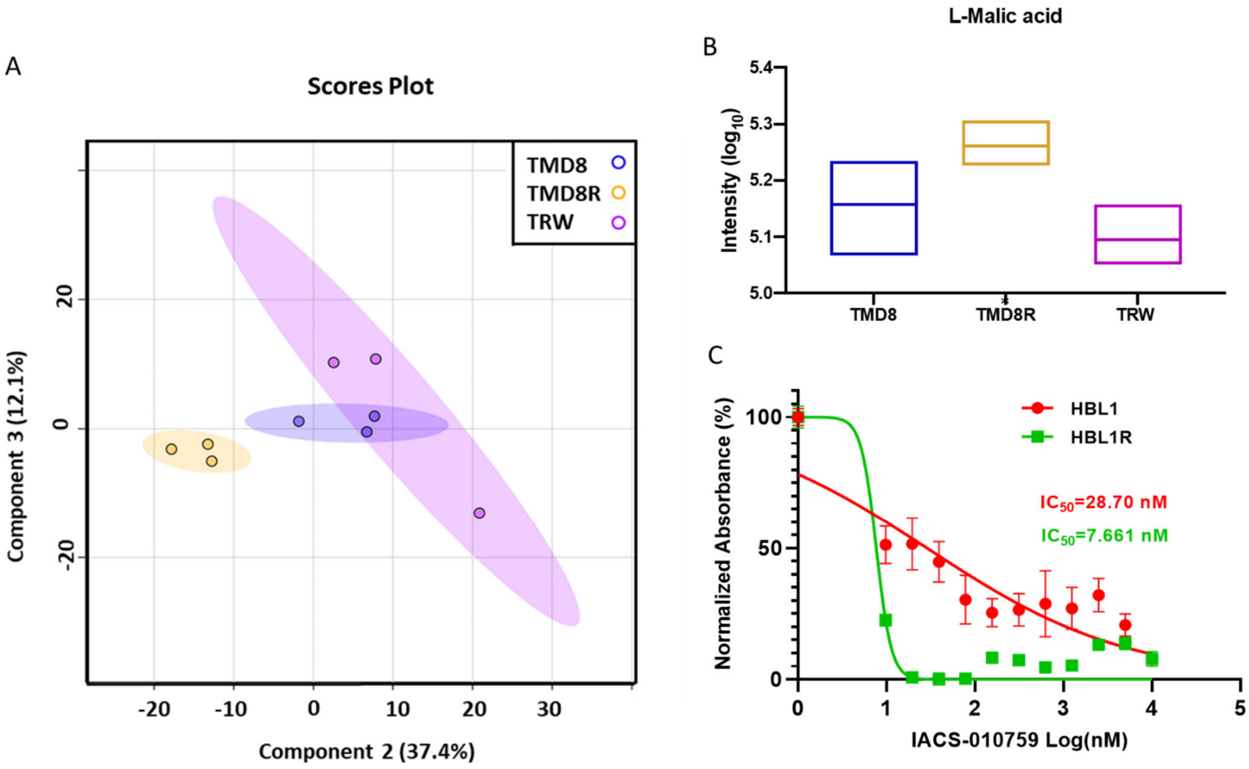

Figure S4. Partial reversal in TMD8R cells upon overexpression of WT BTK gene. A) PLS-DA plot B) Relative abundance change of TCA metabolites in TMD8, TMD8R and TMD8R expressing WT BTK gene. C) IC-50 of the IACS-010759 showing more sensitivity in the ibrutinib resistant HBL1R.

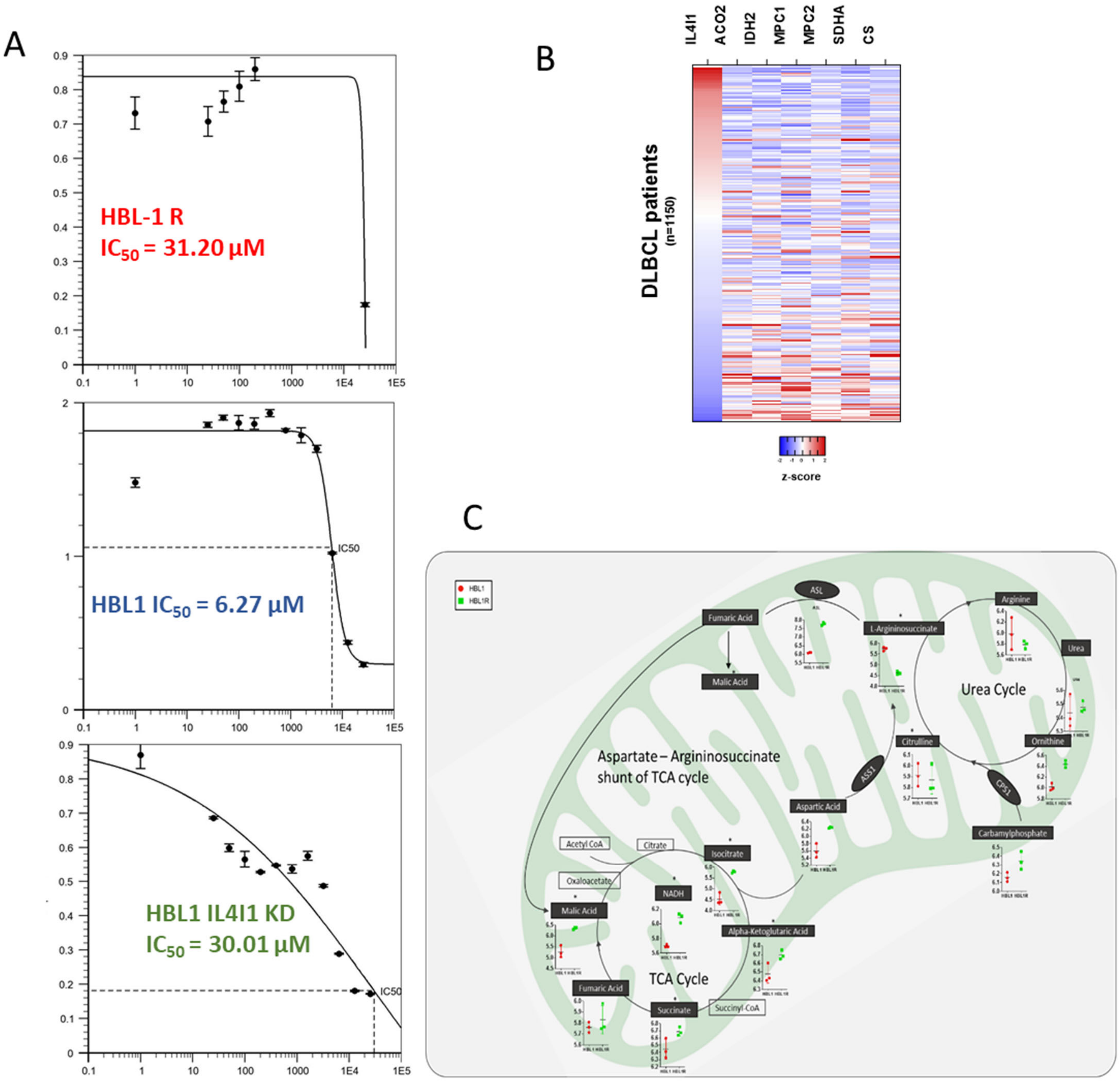

Figure S5. Aspartate-Arginosuccinate shunt of TCA cycle A) Graph depicting IC50 concentrations of the HBL1 cells with either control or IL4I1 siRNA and HBL1 resistant lymphomas treated with Ibrutinib. B)) Correlation heat map along with Z-score of the IL4I1 with the key metabolic genes in the 1150 DLBCL patients C) Aspartate-arginino-succinate shunt at the crosstalk of the TCA and urea cycles constructed based on metabolomics analysis of the HBL1/R cell pair (HBL1 Sens in Red and HBL1R in Green).

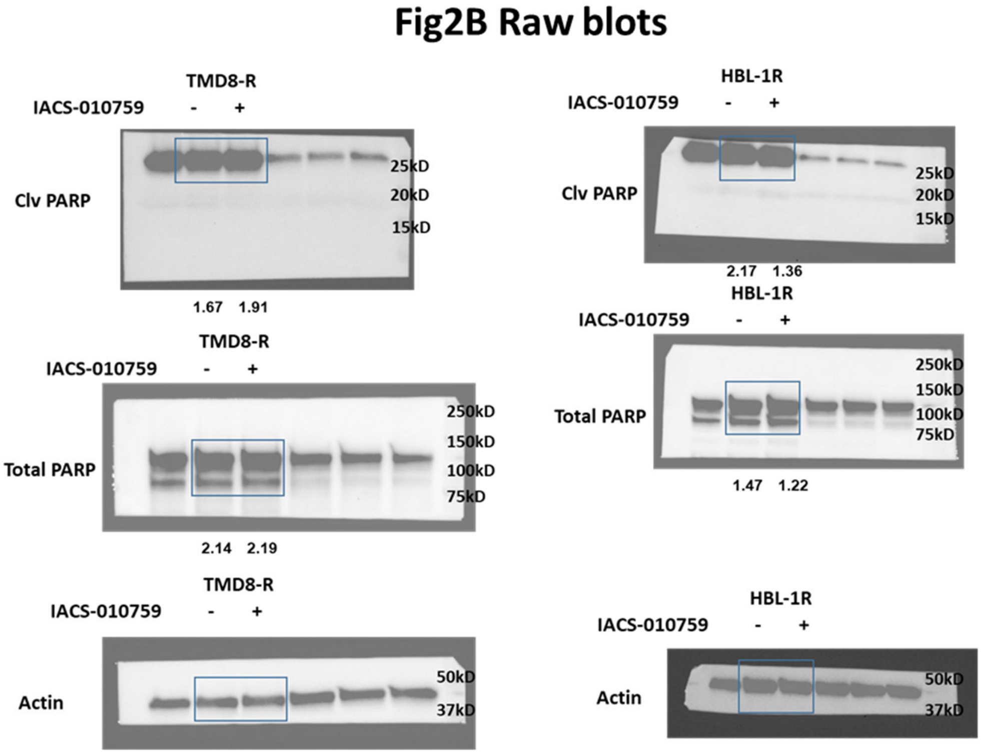

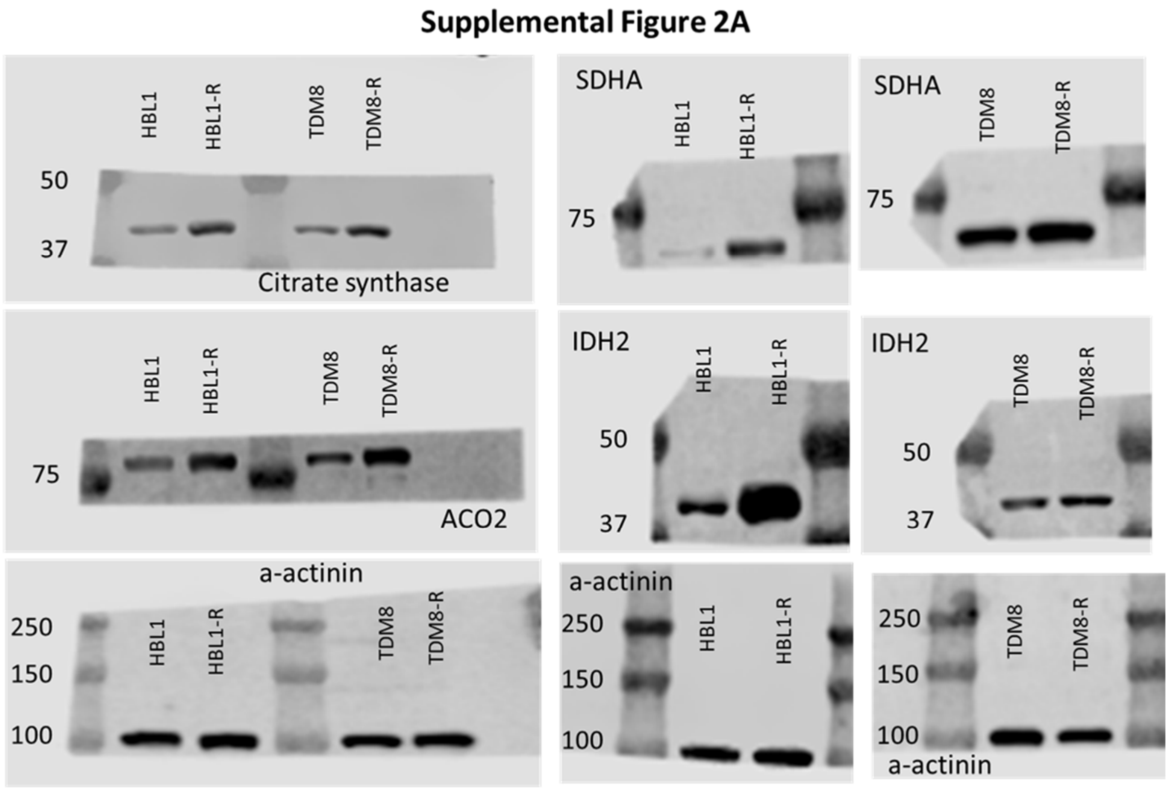

Figure S6. Raw Blots for all the western blots for Figure 2B and SF2A.

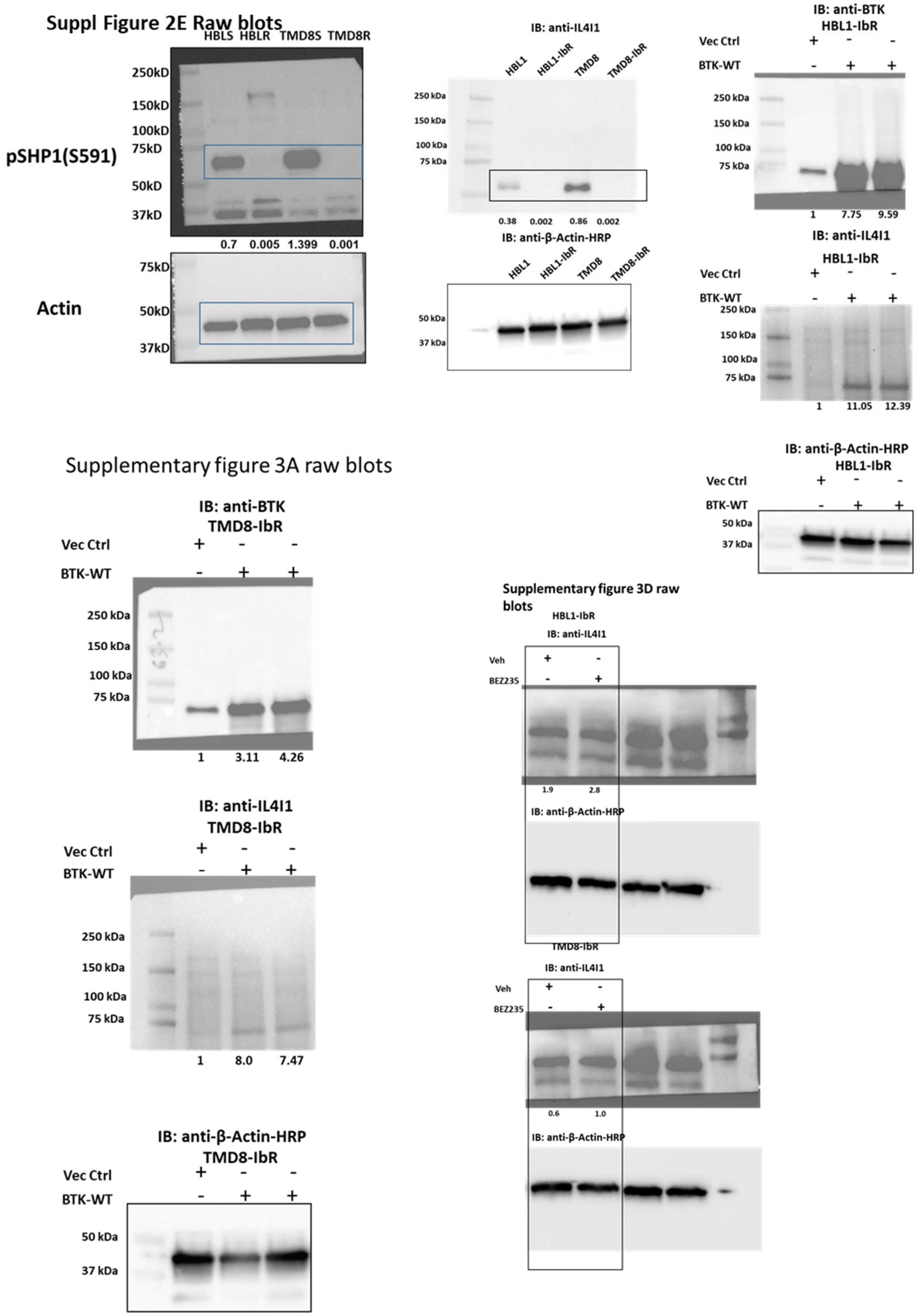

Figure S7. Raw Blots for all the western blots for SF2E, Figure 4A and 4B, SF3A.

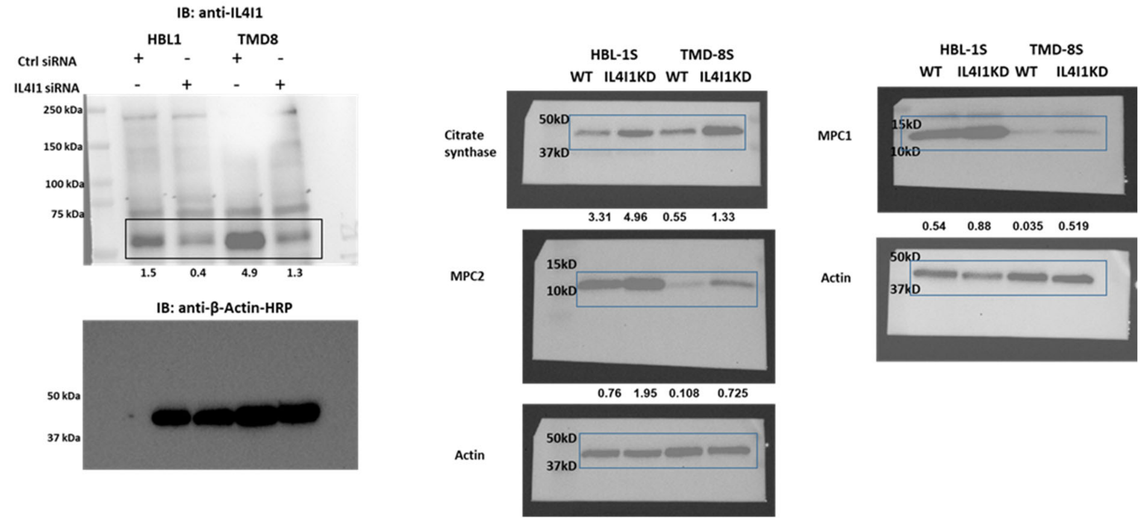

Fig5G Raw blots

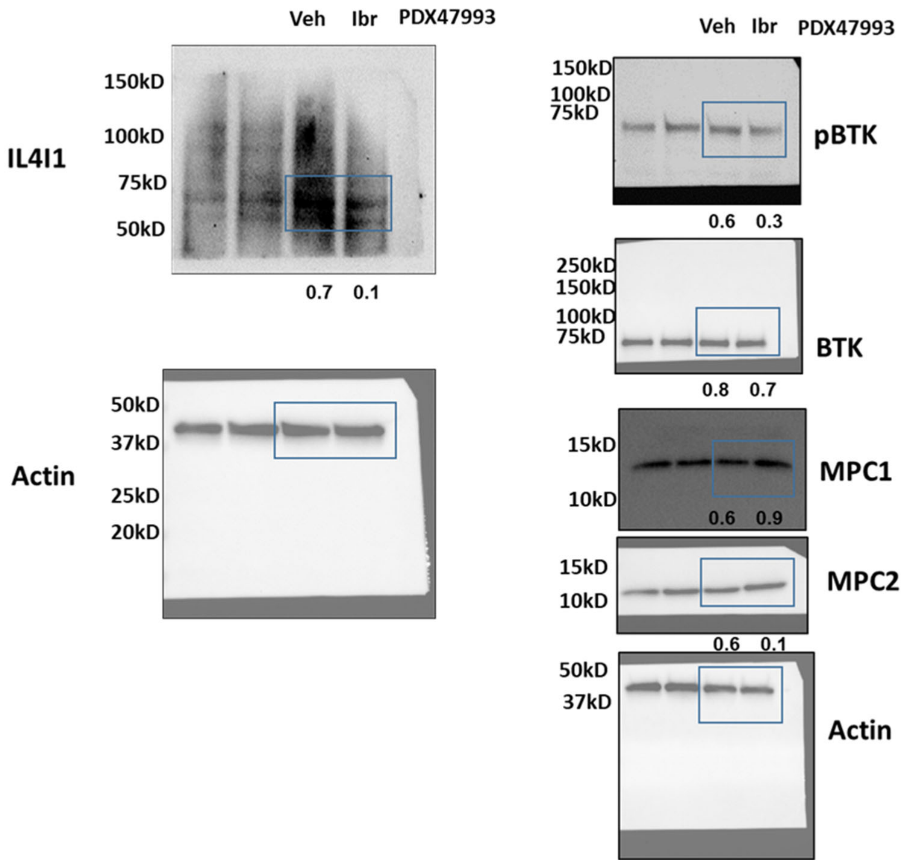

Figure S8. **Raw Blots for all the western blots for Figure 5A, 5E and 5G.**
